# Prognostic value of neutrophil‐to‐lymphocyte ratio and platelet‐to‐lymphocyte ratio for breast cancer patients: An updated meta‐analysis of 17079 individuals

**DOI:** 10.1002/cam4.2281

**Published:** 2019-06-13

**Authors:** Wanying Guo, Xin Lu, Qipeng Liu, Ting Zhang, Peng Li, Weiqiang Qiao, Miao Deng

**Affiliations:** ^1^ Department of Breast Surgery The First Affiliated Hospital, and College of Clinical Medicine of Henan University of Science and Technology Luoyang China; ^2^ Department of Gastrointestinal Surgery The First Affiliated Hospital, and College of Clinical Medicine of Henan University of Science and Technology Luoyang China

**Keywords:** breast cancer, meta‐analysis, neutrophil‐to‐lymphocyte ratio, platelet‐to‐lymphocyte ratio, prognosis

## Abstract

**Aims:**

This study aimed to evaluate the prognostic effect of neutrophil‐to‐lymphocyte ratio (NLR) and platelet‐to‐lymphocyte ratio (PLR) for patients with breast cancer (BC).

**Methods:**

A literature search was performed by searching medical databases. Basic characteristics and prognostic data were extracted from included studies. Primary outcomes, such as overall survival (OS) and disease‐free survival (DFS), were synthesized and compared. Subgroup analyses were performed according to pathology, geographical region, cut‐off value, and tumor progression.

**Results:**

A total of 39 studies comprising 17079 BC patients were included in this meta‐analysis. Among them, 28 studies with 142 64 BC patients investigated predicting role of NLR for OS, showing elevated NLR were associated poor prognosis (hazard ratio [HR]: 1.78, 95% confidence interval [CI]: 1.49‐2.13, *P* < 0.001). Twenty‐seven studies containing 115 04 patients explored the role of NLR in predicting DFS, showing elevated NLR was associated with poor DFS with HR of 1.60 (95% CI: 1.42‐1.96, *P* < 0.001). Twelve studies explored the role of PLR in predicting OS, showing patients with higher PLR were associated with a significantly worse prognosis with a pooled HR of 1.32 (95% CI: 1.11‐1.57, *P* = 0.002). Eleven studies with 5013 patients shown patients with elevated PLR were associated shorter DFS (HR: 1.43, 95% CI: 1.09‐1.86, *P* = 0.009). Subgroup analyses shown a greater magnitude of association between NLR and OS in triple‐negative BC patients than in HER2‐positive ones.

**Conclusions:**

Our study suggested that elevated NLR and PLR were associated with poor OS as well as high risk of recurrence for BC patients. Subgroup analyses confirmed the prognostic effect of NLR and PLR in HER2‐positive BC patients. As easily accessible parameters, NLR and PLR should be identified as useful biomarkers in the management of BC.

## 1 INTRODUCTION

Breast cancer (BC) is the most frequently diagnosed malignance among women and the one of the most common causes of cancer‐related death.^1^, ^2^ Clinical therapeutic strategies and prognosis of BC are based on tumor characteristics, patient factors and response to treatment. However, its high heterogeneity results in a broad range of clinical outcomes even among BC patients with similar clinical staging and pathologic grading.^3^ Tumor microenvironment, inflammation, and immune response have been reported to play important roles in tumor progression and prognosis.^4^, ^5^

Recently, substantial evidence shown that inflammation‐based models, such as the systemic immune‐inflammation index, neutrophil‐to‐lymphocyte ratio (NLR), platelet‐to‐lymphocyte ratio (PLR) and inflammation‐based index, were useful indicators for predicting the prognosis of various solid cancers.^6^, ^7^ NLR and PLR, two of the most frequently applied indicators, have been widely investigated for their value in predicting prognosis of BC patients. A majority of researches demonstrated elevated peripheral NLR and PLR were recognized as poor prognostic factors.^8^, ^9^ Nevertheless, there were still others revealed patients with elevated PLR were associated with better survival outcomes.^10^

Therefore, this study was conducted to evaluate the prognostic value of NLR and PLR on overall survival (OS) and disease‐free survival (DFS) for BC patients. Moreover, by performing subgroup analyses, we quantified the effect of NLR and PLR in different subgroups.

## 2 METHODS

### 2.1 Search strategy

A comprehensive literature search of relevant studies was performed through the online medical databases PubMed, Embase, Web of Science, the Cochrane Library and Scopus. Studies focused on the correlation of BC and NLR as well as PLR were taken into retrievement. Search terms were confined to the following main words and Medical Subject Headings terms: “neutrophil”, “platelet”, “lymphocyte”, “neutrophil‐to‐lymphocyte”, “platelet‐to‐lymphocyte”, “breast cancer”, and “breast carcinoma”. Moreover, by using cross‐references from the references of primary selected studies and relevant studies, a backward search was also performed to ensure a comprehensive search. Literatures were restricted to those written in English. There was no restriction on geographical region. Two reviewers (Wanying Guo and Xin Lu) completed the electronic search independently.

### 2.2 Study inclusion and exclusion criteria

Reviewers screened the eligible studies based upon inclusion and exclusion criteria which were prespecified. The final decision on inclusion and exclusion criteria were approved by all the authors. Any disagreements between the two reviewers were solved and made final decision by the senior reviewer (Miao Deng).

The criteria for inclusion were as following:

1. Articles analyzed BC patients.
2. Articles evaluated prognostic value of NLR or PLR.
3. Articles assessed the OS or DFS of BC patients.

The criteria for exclusion were as following:

1. Articles not focused on the prognosis of BC patients.
2. Articles concerned on neither pretreatment NLR nor PLR.
3. Reviews or editorials.
4. Case reports or conference abstracts.
5. Articles without data of interest (OS or DFS).

Articles with duplicate sample set, such as those published by same authors or departments, were picked out for further screening. In this case, only the articles with largest sample size or those published most recently were finally included in the present meta‐analysis. However, for those studies analyzed two or more independent sample sets such as training and validation cohorts, the cohorts were enrolled in this study and analyzed independently.

### 2.3 Data management and statistical analysis

The software of Endnote (version X8) was used for preliminary screening and sorting. Data were extracted from the enrolled literatures by two authors (Wanying Guo and Xin Lu) after reading full text intensively. The baseline information included full list of authors as well as affiliations, year of publication, geographical region, research centers, models of use, sample size, cut‐off values for NLR and PLR, mean or median ages, proportion of triple negative patients, indications for surgical treatment, follow‐up time, and treatment strategies. The hazard ratios (HRs) with 95% confidential intervals (CIs) were directly extracted from the tables or texts. However, in several studies, the HRs and 95% CIs were not shown directly. In such cases, the software of Engauge Digitizer (version 4.1) was used to extract HRs and 95% CIs by computing the Kaplan‐Meier graph.^11^, ^12^ The primary outcomes were pooled using the Cochrane Collaboration's Review Manager (version 5.3, Cochrane Collaboration, Oxford, UK).^13^ Random effect model was applied routinely only if no obvious heterogeneity was observed among the included literatures (*I* ^2^ < 40%). Heterogeneity within studies was explored by using the chi‐square test with a P value of 0.10 for significance. Moreover, the heterogeneities were quantified using the *I* ^2^ statistics. Sensitivity analyses of main outcomes were conducted by using the software of Stata (version 12.0).^14^ The publication bias was investigated using funnel plots. Moreover, the symmetry properties of funnel plot was examined by using Begg and Egger tests.^15^

### 2.4 Risk of bias assessment

All the included studies were critically assessed for methodological quality by 2 researchers independently (Lu X and Guo WY) by using the Quality In Prognosis Studies tool. Each study was graded for the following domains: study participation, study attrition, prognostic factor measurement, outcome measurement, study confounding, and statistical analysis and reporting. The risk of bias for each domain is graded as low (−), moderate (±), or high (+).

### 2.5 Subgroup analysis

To identify the sources of heterogeneity, subgroup analyses were conducted according to etiologies, geographical region, cut‐off values, and tumor stage. Subgroup of triple‐negative included BC patients with negatively expressed estrogen receptor, progesterone receptor, and HER2, while subgroup of HER2‐positve enrolled BC patients with positive HER2 expression. Patients from different continents were classified into Asia, America, and Europe subgroups respectively. Patients with early stage and metastatic tumors were analyzed in different subgroups. It was difficult to subgroup analysis which based on therapeutic strategies because of most of BC patients received surgical treatment as well as other treatments like adjuvant chemotherapy, endocrine therapy or trastuzumab therapy.

## 3 RESULTS

### 3.1 Characteristics of included studies

**Figure 1:**
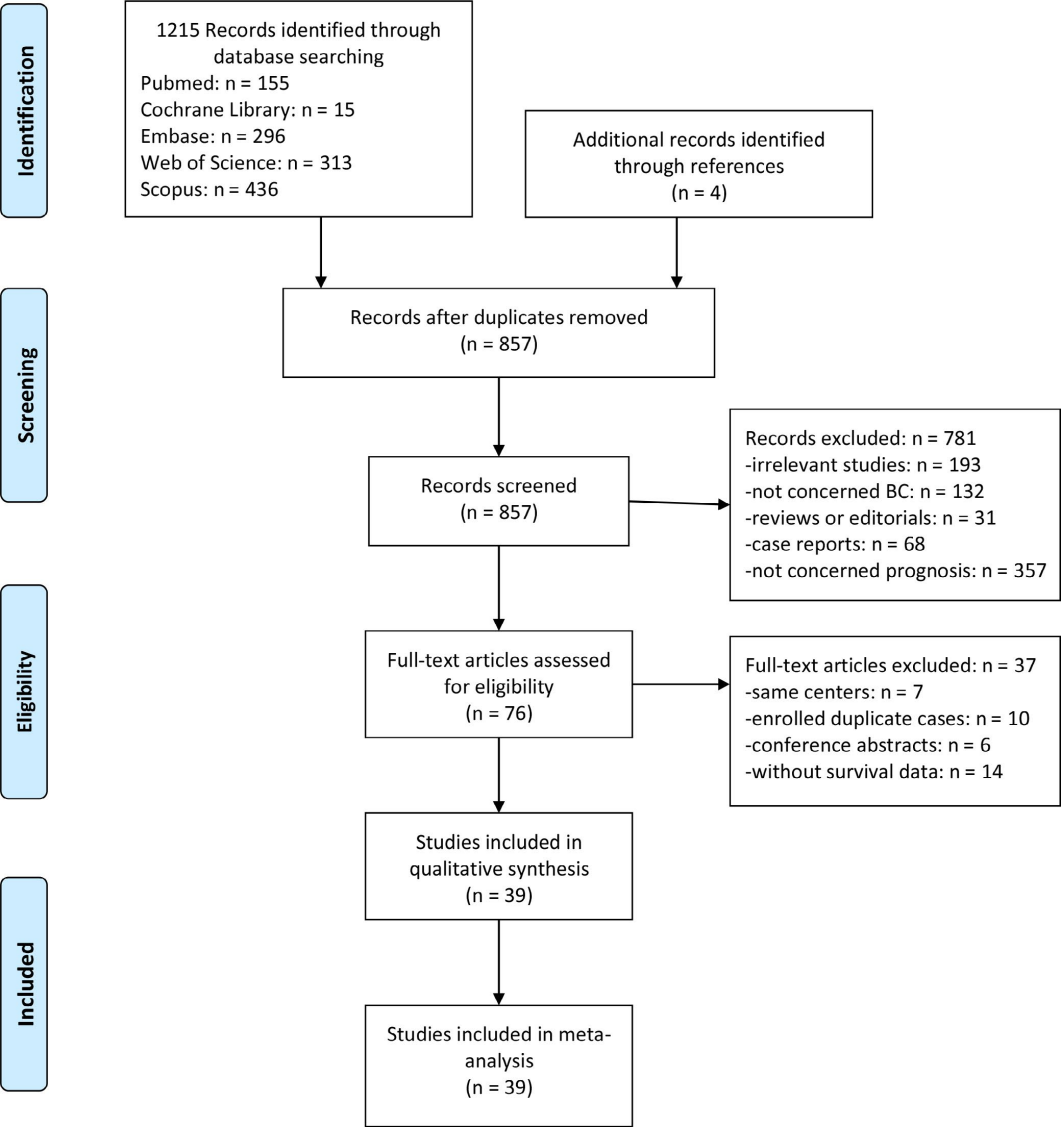
Flow diagram showing study retrieval and selection process.

Literature research identified 1215 records: 115 from PubMed, 296 from Embase, 313 from Web of Science, 15 from the Cochrane Library, and 436 from Scopus. Meanwhile, 4 studies were identified through references. As shown in Figure 1, after screening titles and abstracts, 362 duplications and an additional 781 studies were excluded. Full text of the remaining 76 studies were read rigorously. Thirty‐seven more were excluded: 7 were published by same centers, 10 enrolled duplicate patients, 6 were conference abstracts and 14 were without survival data. Finally, a total of 39 studies with 17079 patients were included in the present meta‐analysis.^8^, ^9^, ^10^, ^16^, ^17^, ^18^, ^19^, ^20^, ^21^, ^22^, ^23^, ^24^, ^25^, ^26^, ^27^, ^28^, ^29^, ^30^, ^31^, ^32^, ^33^, ^34^, ^35^, ^36^, ^37^, ^38^, ^39^, ^40^, ^41^, ^42^, ^43^, ^44^, ^45^, ^46^, ^47^, ^48^, ^49^, ^50^ Among them, 35 studies with 159 39 BC patients analyzed the effectiveness of NLR and 15 studies with 7949 patients analyzed PLR. The characteristics of the 39 included studies were summarized in Table 1.

**Table 1:** Characteristics of included studies

Study	Year	Country	Design, center	Models	Endpoint	HRs	Sample size	Cut‐off	Ages (Years)	Triple negative tumors (%)	Diagnosis	Follow‐up (months)	Therapies
Allan et al	2016	Costa Rica	Retrospective, single center	NLR & PLR	OS & DFS	Reported	172	NLR: 3; PLR: 250	54.2 ± 12.7	34 (19.6)	Nonmetastatic breast cancer	79.3 (2‐90)	Adjuvant chemotherapy
Asano et al	2015	Japan	Retrospective, single center	NLR	OS & DFS	Reported	177	NLR: 3	≤56:87;>56:90	61 (34.5)	Early stage breast cancer	40.8 (7.2‐72)	Neoadjuvant chemotherapy
Asano et al	2016	Japan	Retrospective, single center	PLR	OS & DFS	Reported	177	PLR: 150	≤56:87;>56:90	61 (34.5)	Early stage breast cancer	37.2 (1.2‐72)	Neoadjuvant chemotherapy + surgery
Azab et al	2013	USA	Retrospective, single center	NLR & PLR	OS	Reported	437	NLR: 3.3; PLR: 185	63.6 ± 0.7	NA	Breast cancer with all stages	Mean: 60	Surgery or chemotherapy or radiotherapy
Blanchette et al	2018	Canada	Retrospective, single center	PLR	OS & FFS	Reported	154	PLR: 185	56 (47‐63)	0 (0)	HER2‐positive metastatic breast cancer	34 (6‐124)	Trastuzumab therapy
Bozkurt et al	2015	Turkey	Retrospective, single center	NLR	OS & DFS	Reported	85	NLR: 2	≤50:57;>50:28	85 (100)	Early stage breast cancer	NA	Surgery + adjuvant chemotherapy
Chae et al	2018	Korea	Retrospective, single center	NLR	DFS	Reported	87	NLR: 1.7	45.8 ± 11.2	87 (100)	Early stage breast cancer	Median: 57	Neoadjuvant chemotherapy + surgery
Chen et al	2016	China	Retrospective, single center	NLR	DFS & CSS	Reported	215	NLR: 2.1	46.41 ± 9.82	18 (8.4)	TNM stage II‐III	57.6 ± 27.1	Neoadjuvant chemotherapy + surgery
Cho et al	2018	Korea	Retrospective, single center	NLR & PLR	DFS & CSS	Reported	661	NLR: 1.34; PLR: 185.5	52.7 ± 11.5	117 (17.7)	All TNM stages	72 (1‐189)	Surgery
Cihan et al	2014	Turkey	Retrospective, single center	NLR & PLR	OS & DFS	Reported	350	NLR: 3; PLR: 160	55.3 ± 0.3	NA	All TNM stages	0.3‐112	Surgery + adjuvant therapies
Dirican et al	2015	Turkey	Retrospective, single center	NLR	OS & DFS	Reported	1527	NLR: 4	≤35:92;36‐50:619;>50:816	214 (14)	All TNM stages	30 (2‐75)	Surgery
Ferroni et al	2018	Italy	Retrospective, Database	NLR	OS & DFS	Reported	475	NLR: 2	57 ± 13	57 (12)	All TNM stages	45.6 (3.12‐139.2)	Surgery
Forget et al	2014	Belgium	Retrospective, Database	NLR	OS & DFS	Reported	425	NLR: 3.3	25‐89	NA	Resectable breast cancer	69.8 (53.5‐89.9)	Surgery
Gunduz et al	2015	Turkey	Retrospective, single center	PLR	DFS	Reported	62	PLR: 200	52 (24‐73)	NA	Advanced breast cancer	Median: 48.4	Surgery + adjuvant chemotherapy
Hernandez et al	2017	Spain	Retrospective, single center	NLR	OS & DFS	Reported	150	NLR: 3.3	49.8 (28‐77)	38 (25.3)	TNM stage I‐III	24 (1‐144)	Neoadjuvant chemotherapy + surgery
Hong et al	2016	China	Retrospective, single center	NLR	OS & DFS	Reported	487	NLR: 1.93	55 (28‐89)	94 (19.2)	Primary invasive breast cancer	Median: 55	Surgery
Iwase et al	2017	Japan	Retrospective, single center	NLR	OS	Reported	89	NLR: 3	50.9 ± 11.3	24 (27)	Recurrent breast cancer	NA	Chemotherapy
Jia et al	2015	China	Retrospective, single center	NLR	OS & DFS	Reported	1570	NLR: 2	48.9 ± 11.8	225 (14.3)	Operable breast cancer	79 (4‐172)	Surgery
Koh et al	2014	Korea	Retrospective, single center	NLR	OS & DFS	Reported	157	NLR: 2.25	44 (24‐71)	0 (0)	ER/PR(+) and HER2(−)	21 (1‐108)	Neoadjuvant chemotherapy + surgery
Koh et al	2015	Malaysia	Retrospective, single center	NLR & PLR	OS& DFS	Reported	1435	NLR: 4; PLR: 185	Median: 52	208 (14.5)	All TNM stages	NA	Surgery or chemotherapy or radiotherapy
Krenn‐Pilko et al	2014	Austria	Retrospective, single center	PLR	OS & DFS	Reported	747	PLR: 292	57.9 ± 12.2	**NA**	Nonmetastatic breast cancer	98 ± 29.2	Surgery
Krenn‐Pilko et al	2016	Austria	Retrospective, single center	NLR	OS & DFS	Reported	747	NLR: 3	58.1 ± 12.2	**NA**	Nonmetastatic breast cancer	Median: 106	Surgery
Lee et al	2018	Korea	Retrospective, single center	NLR	OS & DFS	Reported	358	NLR: 3.16	Median: 51	358 (100)	Advanced triple‐negative breast cancer	NA	Surgery
Limori et al	2018	Japan	Retrospective, single center	NLR	OS	Reported	34	NLR: 3	63 (44‐88)	NA	Stage IV breast cancer	NA	Endocrine therapy
Liu et al	2016	China	Retrospective, single center	NLR & PLR	OS & DFS	Reported	318	NLR: 3; PLR: 147	45 (19‐71)	161 (50.6)	HR(−) nonmetastatic breast cancer	58.1 (5.9‐136.1)	Surgery
Mando et al	2018	Argentina	Retrospective, single center	NLR	DFS	Reported	85	NLR: 2	56 (44‐66)	5 (5.9)	Early stage breast cancer	38.6 (29.4‐60.1)	Surgery
Miyagawa et al	2018	Japan	Retrospective, single center	NLR	OS	Reported	59	NLR: 3	34‐83	12 (21.4)	Metastatic breast cancer	NA	Chemotherapy
Nakano et al	2014	Japan	Retrospective, single center	NLR	DFS & CSS	Reported	167	NLR: 2.5	57.9 ± 10.9	NA	Operable breast cancer with stage I‐III	85.8 (19.8‐148.9)	Neoadjuvant chemotherapy or surgery
Orditura et al	2016	Italy	Retrospective, single center	NLR	DFS	Reported	300	NLR: 1.97	≤35:9;>35:291	30 (10)	Early stage breast cancer	Median: 84	Surgery
Pistelli et al	2014	Italy	Retrospective, single center	NLR	OS & DFS	Reported	90	NLR: 3	53 (28‐79)	90 (100)	Early stage breast cancer	53.8 (13.1‐95.2)	Surgery
Qiu et al	2018	China	Retrospective, single center	NLR	OS & DFS	Reported	406	NLR: 2.85	44 (21‐75)	406 (100)	Early stage breast cancer	54.3 (7.8‐126.5)	Surgery + chemotherapy
Takeuchi et al	2017	Japan	Retrospective, single center	NLR & PLR	DFS	Reported	296	NLR: 2.06; PLR: 162.28	<50:61;≥50:235	NA	Localized breast cancer	Median: 41	Surgery
Takuwa et al	2018	Japan	Retrospective, single center	NLR	OS	Reported	171	NLR: 1.9	59 (31‐89)	NA	Metastatic breast cancer	44 (0‐217)	Multidisciplinary therapy
Templeton et al	2018	Spain	Prospective, 65 GEICAM institutions	NLR	OS & DFS	Reported	1243	NLR: 1.35	50 (23‐76)	107 (8.6)	Early stage breast cancer	Median: 120	Surgery + chemotherapy
Ulas et al	2015	Turkey	Retrospective, two centers	NLR & PLR	OS & DFS	Reported	51	NLR: 2.38; PLR: 161.28	51.4 ± 10.4	NA	HER2‐positive early breast cancer	26 (6‐84)	Surgery + adjuvant chemotherapy
Vernieri et al	2018	Italy	Retrospective, single center	NLR & PLR	OS	Reported	57	NLR: 2.5; PLR: 200	56 (33.7‐78.9)	57 (100)	Metastatic breast cancer	NA	Chemotherapy
Wariss et al	2017	Brazil	Retrospective, one center	NLR & PLR	OS	Reported	2288	NLR: 4; PLR: 150	55 (18‐98)	301 (12.7)	All TNM stages	NA	All kinds of therapies
Yao et al	2014	China	Retrospective, one center	NLR & PLR	OS & DFS	Reported	608	NLR: 2.56; PLR: 107.64	52.4 ± 10.8	98 (17.2)	Operable breast cancer	42 (8‐62)	Surgery
Zhang et al	2016	China	Retrospective, one center	NLR	OS & DFS	Reported	162	NLR: 1.81	50.8 ± 10.6	NA	TNM stage I‐III	NA	Surgery

Note

CSS, cancer‐specific survival; DFS, disease‐free survival; FFS, failure‐free survival; HRs, hazard ratios; N/A, not available; NLR, neutrophil‐to‐lymphocyte ratio; OS, overall survival; PLR, platelet‐to‐lymphocyte ratio; TNM, Tumor, node, metastases; GEICAM, Spanish Group for the Investigation of Breast Cancer Ages and Follow‐up periods were expressed as mean ± SD or median (range).

The bolds represent summary of subgroup analyses for OS and DFS.

### 3.2 Overall and subgroup analysis for NLR

**Figure 2:**
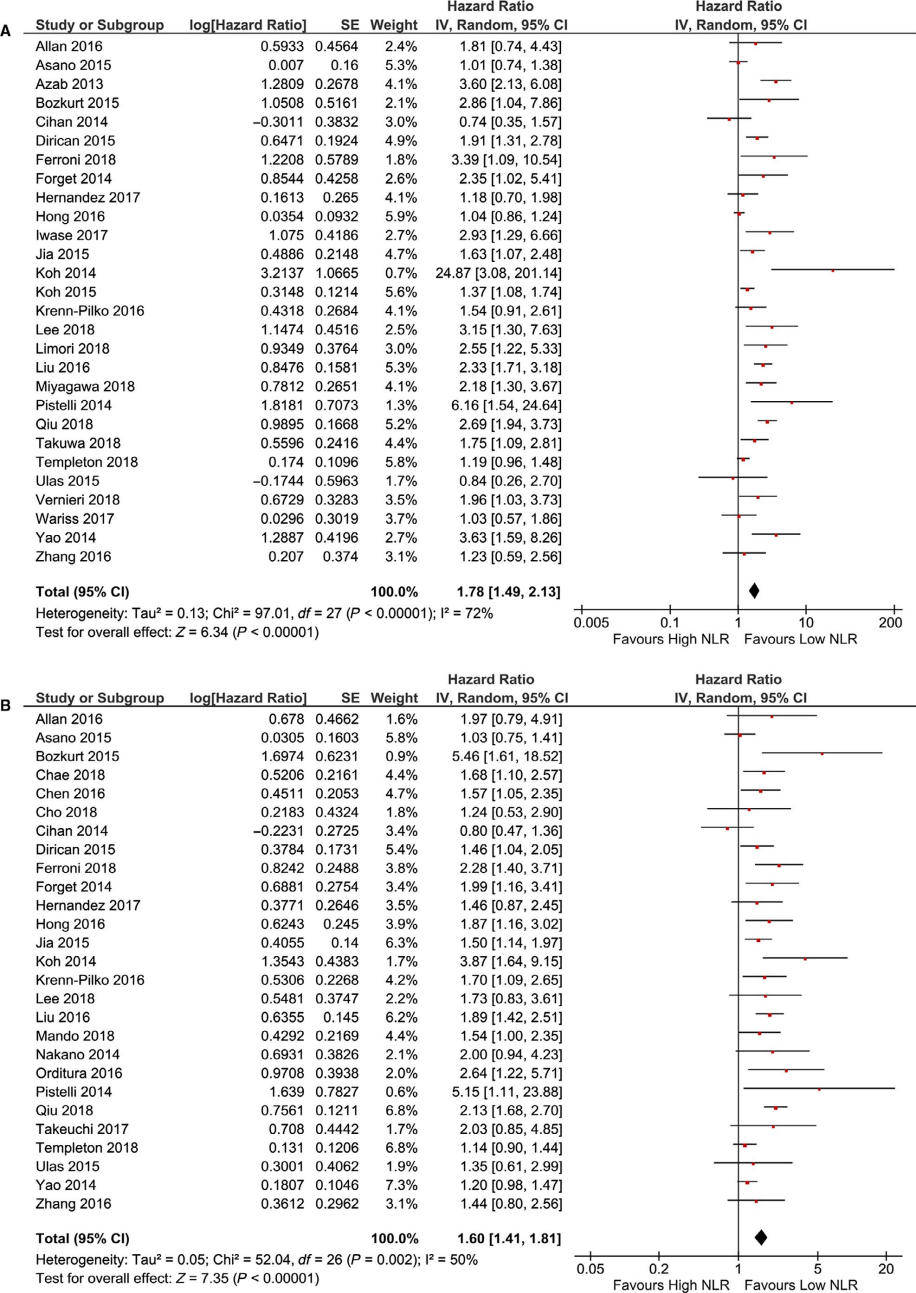
Forest plot of NLR in predicting OS (a) and DFS (b) of BC patients.

Twenty‐eight studies comprising 142 64 BC patients investigated predicting role of NLR for OS. The pooled results shown that patients with elevated NLR prior treatment were associated worse prognosis compared to those with lower NLR (HR: 1.78; 95% CI: 1.49‐2.13; *P* < 0.001). Twenty‐seven cohort studies containing 115 04 patients explored the prognostic role of NLR in predicting DFS. The results demonstrated that patients with elevated NLR were associated with poor DFS, with a HR of 1.60 (95% CI: 1.42‐1.96; *P* < 0.001) (Figure 2).

Results for subgroup analyses were shown in Table 2. The results of other subgroups displayed similar outcomes to overall result except subgroup of HER2‐positive and America. Six studies analyzed patients with HER2‐positively expressed showed that NLR was not significantly associated with OS (HR: 1.27; 95% CI: 0.94‐1.72; *P* = 0.12). In subgroup of America, the pooled result also showed no significant correlation between NLR with OS (HR: 1.91; 95% CI: 0.83‐4.37; *P* = 0.13). The results of subgroups for predicting DFS were similar to overall result. However, subgroup analyses based on tumor progression was unable to conduct due to insufficient data.

**Table 2:** Subgroup analyses of NLR for OS and DFS

Subgroups	Independent cohorts	Sample size	HR* (95% CI) (H/L*)	*P* value	Study heterogeneity			
					*χ* ^2^	df*	*I* ^2^, %	*P* value
**Overall survival**	**28**	**142 64**	**1.78 (1.49‐2.13)**	**<0.0001**	**97.01**	**27**	**72**	**<0.0001**
Pathology								
Triple‐negative	12	2157	2.18 (1.75‐2.73)	<0.0001	15.70	11	30	0.15
Her2‐positive	6	1094	1.27 (0.94‐1.72)	0.12	8.19	5	39	0.15
Cut‐off value								
<3	12	5608	1.79 (1.33‐2.41)	0.0001	47.85	11	77	<0.0001
≥3	16	8656	1.79 (1.42‐2.24)	<0.0001	44.85	15	67	<0.0001
Tumor progression								
Early‐stage	16	6984	1.70 (1.33‐2.18)	<0.0001	65.10	15	77	<0.0001
Metastatic	6	768	2.17 (1.68‐2.81)	<0.0001	2.27	5	0	0.81
Geographical region								
Asia	18	8180	1.79 (1.42‐2.25)	<0.0001	71.50	17	76	<0.0001
America	3	2897	1.91 (0.83‐4.37)	0.13	9.69	2	79	0.008
Europe	7	3187	1.65 (1.20‐2.27)	0.002	11.94	6	50	0.06
**Disease‐free survival**	**27**	**115 04**	**1.60 (1.42‐1.96)**	**<0.0001**				
Pathology								
Triple‐negative	10	1674	1.77 (1.44‐2.18)	<0.0001	14.40	9	38	0.11
Her2‐positive	5	881	1.41 (1.05‐1.89)	0.02	8.66	4	54	0.07
Cut‐off value								
<3	17	7190	1.67 (1.42‐1.96)	<0.0001	34.16	16	53	0.005
≥3	10	4313	1.50 (1.21‐1.86)	0.0003	17.63	9	49	0.04
Geographical region								
Asia	18	7817	1.57 (1.34‐1.83)	<0.0001	37.75	17	55	0.003
America	2	257	1.61 (1.09‐2.85)	0.02	0.23	1	0	0.63
Europe	7	3430	1.75 (1.30‐2.35)	0.0002	13.92	6	57	0.03

Abbreviations: CI*, confidence interval; df*, degrees of freedom; HR, Hazard Ratio; H, High group, L, Low group.

The bolds represent summary of subgroup analyses for OS and DFS.

### 3.3 Overall and subgroup analyses for PLR

**Figure 3:**
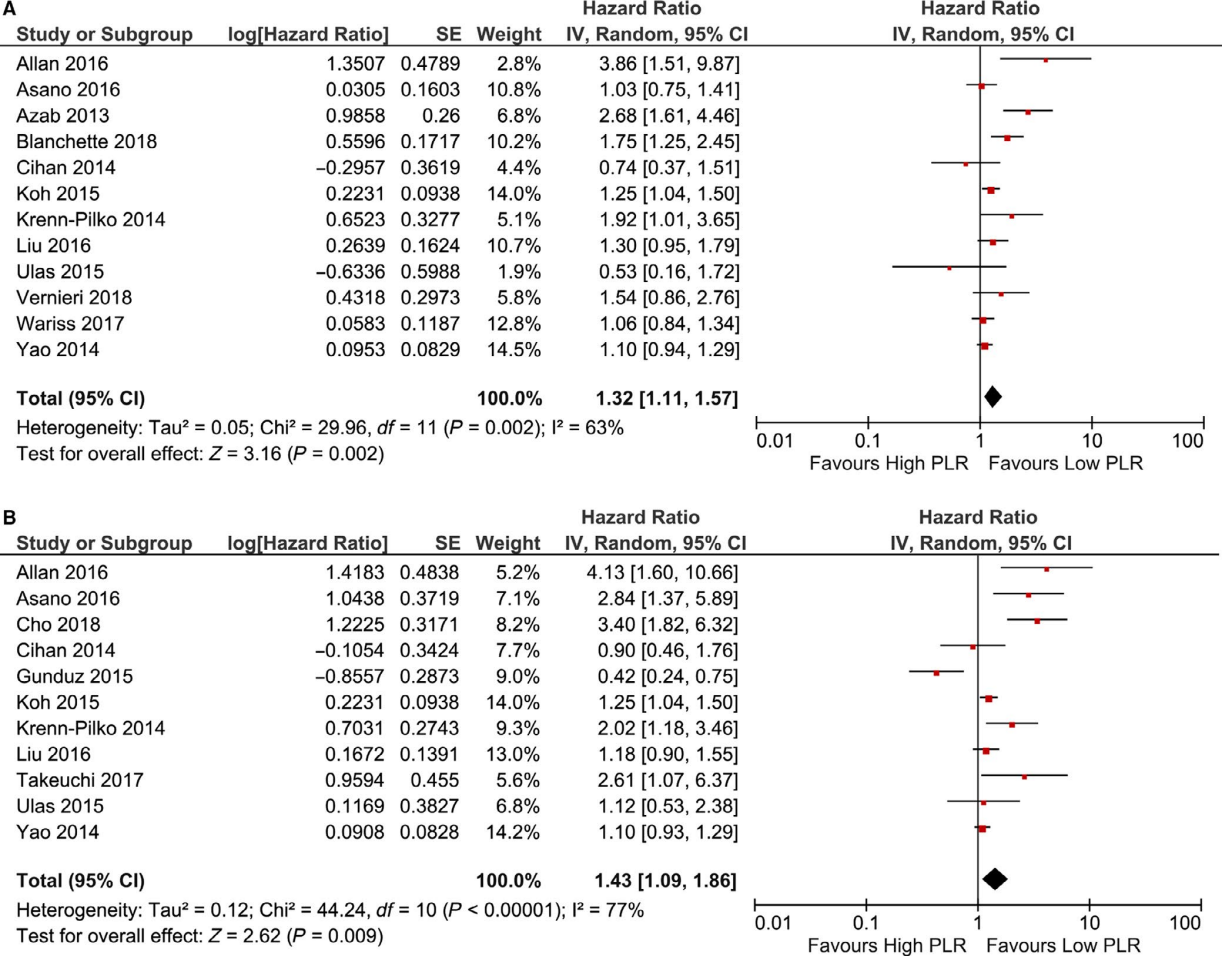
Forest plot of PLR in predicting OS (a) and DFS (b) of BC patients.

As shown in Table 3, 12 studies with 6930 patients explored the prognostic role of PLR in predicting OS of patients with BC. The pooled outcome suggested that patients with higher PLR were associated with a significantly poor prognosis (HR: 1.32; 95% CI: 1.11‐1.57; *P* = 0.002). Eleven studies with 5013 patients investigated predicting role of PLR for DFS shown that patients with elevated PLR were associated with shorter DFS (HR: 1.43; 95% CI: 1.09‐1.86; *P* = 0.009) (Figure 3).

**Table 3:** Subgroup analyses of PLR for OS and DFS

Subgroups	Independent cohorts	Sample size	HR* (95% CI) (H/L*)	*P* value	Study heterogeneity			
					*χ* ^2^	df*	*I* ^2^, %	*P* value
**Overall survival**	**12**	**6930**	**1.32 (1.11‐1.57)**	**0.002**				
Pathology								
Triple‐negative	4	727	1.54 (1.03‐2.33)	0.04	9.65	3	69	0.02
Her2‐positive	5	894	1.18 (0.83‐1.70)	0.36	9.70	4	59	0.05
Cut‐off value								
<185	6	3928	1.09 (0.97‐1.22)	0.15	3.94	5	0	0.56
≥185	6	3002	1.81 (1.33‐2.46)	0.0001	14.04	5	64	0.02
Tumor progression								
Early‐stage	6	2209	1.26 (0.97‐1.63)	0.08	11.83	5	58	0.04
Metastatic	2	211	1.69 (1.27‐2.27)	0.0004	0.14	1	0	0.71
Geographical region								
Asia	6	3075	1.14 (1.02‐1.28)	0.02	5.23	5	4	0.39
America	4	3051	1.89 (1.13‐3.14)	0.01	17.88	3	83	0.0005
Europe	2	804	1.70 (1.10‐2.62)	0.02	0.25	1	0	0.62
**Disease‐free survival**	**11**	**5013**	**1.43 (1.09‐1.86)**	**0.009**	**44.24**	**10**	**77**	**<0.0001**
Pathology								
Triple‐negative	1	161	1.40 (0.97‐2.00)	0.07	NA	NA	NA	NA
Her2‐positive	3	406	0.74 (0.42‐1.31)	0.30	5.75	2	65	0.06
Cut‐off value								
<185	6	1936	1.28 (0.99‐1.66)	0.06	10.03	5	50	0.07
≥185	5	3077	1.63 (0.86‐3.07)	0.13	32.74	4	88	<0.0001
Geographical region								
Asia	9	4094	1.28 (0.98‐1.68)	0.07	34.19	8	77	<0.0001
America	1	172	4.13 (1.60‐10.66)	0.003	N/A	N/A	N/A	N/A
Europe	1	747	2.02 (1.18‐3.46)	0.01	N/A	N/A	N/A	N/A

Abbreviations: CI*, confidence interval; df*, degrees of freedom; HR, Hazard Ratio; H, High group, L, Low group; N/A, not available.

The bolds represent summary of subgroup analyses for OS and DFS.

Subgroup analyses shown there were no statistically significant correlation between PLR and OS in patients with HER2‐positive expression or patients with early stage tumor. Subgroup analysis based on cut‐off value suggested a value that more than 185 was better for PLR in predicting prognosis of BC patients (HR: 1.81; 95% CI: 1.33‐2.46; *P* < 0.001). In subgroup analyses for PLR in predicting DFS, no significant association was found (Table 3).

### 3.4 Sensitivity analyses and publication bias

**Figure 4:**
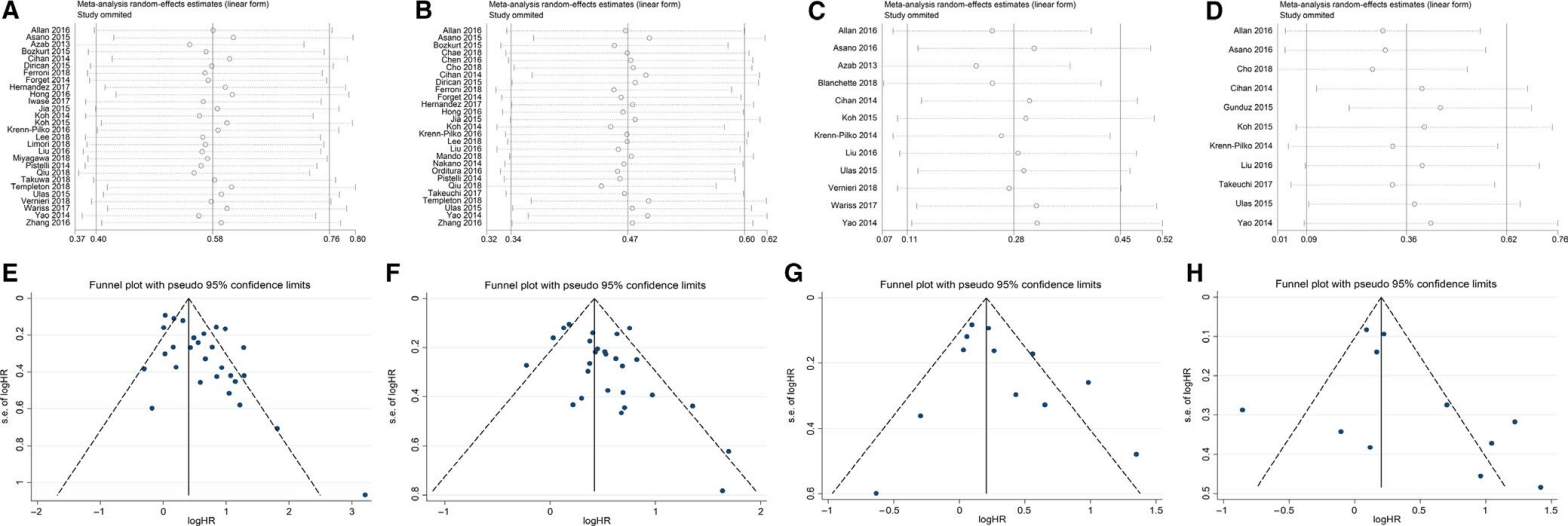
Sensitivity analyses and funnel plots of OS and DFS for NLR (a, b and e, f) and PLR (c, d and g, h).

The sensitivity analyses was conducted to investigate the stabilities of the pooled HRs of OS and DFS by omitting enrolled studies in turn. The results showed that the pooled HRs did not alter significantly after eliminating the included studies in sequence which suggested the steady of our findings (Figure 4).

The funnel plots showed no obvious publication bias among the enrolled studies (studies distributed around the center line symmetrically). Moreover, 2 kinds of statistical tests further validated the dissymmetry of the funnel plot by using Egger's test and Begg's test (Table 4).

**Table 4:** Assessment for publication bias

	Number of estimate	Z value	*P* for Begg's test	t	*P* for Egger's test
NLR for OS	28	1.10	0.272	1.74	0.096
NLR for DFS	27	1.66	0.097	1.20	0.241
PLR for OS	12	1.03	0.304	1.31	0.220
PLR for DFS	11	1.40	0.161	1.38	0.202

Abbreviations: DFS, disease‐free survival; NLR, neutrophil‐to‐lymphocyte ratio; OS, overall survival; PLR, platelet‐to‐lymphocyte ratio.

## 4 DISCUSSION

Recently, more and more studies focused on correlation between inflammation and solid malignancies revealed that tumor initiation, progression, and metastasis were affected by host systemic inflammatory response as well as tumor microenvironment.^4^, ^51^, ^52^ Hence, we performed a meta‐analysis, including 39 studies comprising 17079 BC patients, to evaluate the prognostic role of NLR and PLR in predicting OS and DFS. A significant prognostic effect for NLR and PLR was found on OS and DFS after pooling results. High NLR level was associated with both poor OS as well as DFS for BC patients. Meanwhile, patients with elevated PLR were associated worse OS as well as higher risk of recurrence compared to those with decreased PLR level.

Although the underlying molecular mechanisms have not been adequately illuminated. Neutrophil was considered to be related to cancer‐associated inflammation with the potential mechanism of responding to the ectopic interleukin‐8 released in tumor proliferation, progression and metastasis.^53^ Moreover, cancer‐associated cytokines like tumor necrosis factor‐α and interleukin‐6 contribute to neutrophilia in solid cancers.^54^, ^55^ Neutrophilia inhibits the cytotoxic activity of immune cells like lymphocytes, natural killer cells and T cells which would counteract the anti‐tumor immune response.^56^, ^57^ A high platelet counts was considered to be related to metastasis of BC cells with the mechanism of contribution to lysophosphatidic acid‐dependent metastasis.^58^ Platelets could also promote tumor angiogenesis and stroma formation by secreting vascular endothelial growth factor and facilitating migration of inflammatory cells.^59^, ^60^ Loi et al found elevated lymphocytic infiltration in BC was associated with favorable prognosis, especially in those node‐positive and HER2‐negative BC.^61^ The lymphocytes played an important role in cell‐mediated anti‐tumor immune responses and tumor immunological surveillance.^62^, ^63^, ^64^

In view of the heterogeneity of BC, subgroup analyses according to different subtypes like triple‐negative and HER2‐positive. The results shown a greater magnitude of association between NLR and OS in triple‐negative BC patients than in HER2‐positive ones. And negative prognostic effect was found for NLR and PLR in HER2‐positive BC patients. A previous meta‐analysis performed by Zhang et al included 11 studies and 1 conference abstract to evaluate the prognostic value of PLR in BC.^65^ Their process of extracting data were not rigorous enough that several HRs with 95% were not consistent with original researches.^10^, ^47^ The pooled results of our study were more credible and stable because of more rigorous in data extraction and subgroup analyses. Nonetheless, future prospective studies with large sample size were in need to confirm our outcomes especially the prognostic effect of NLR and PLR on HER2‐positive BC patients. Our subgroup analyses also suggested that a cut‐off value no less than 185 for PLR in predicting OS was more preferable.

Individualized therapy based on tumor‐associated biological characteristics and host circumstance was advocated in treatment strategies for BC. Several treatments such as surgical resection combined with adjuvant chemotherapy or neoadjuvant chemotherapy, endocrine therapy, and targeted therapy were optional for BC patients with different tumor stages. This study performed subgroup analysis based on tumor stage suggested comparative effect of NLR and PLR in predicting OS. The data were insufficient to conduct subgroup analysis based on treatment strategies.

To our knowledge, this was the most comprehensive meta‐analysis with largest sample size to estimate the prognostic role of PLR as well as NLR for BC. However, there were still several limitations should be taken into consideration when interpreting our findings. First, although there was no obvious publication bias, all the included studies were retrospectively designed. High proportion of retrospective individual studies would give rise to inherent bias inevitably. It would be preferable for future studies to design and collect data prospectively. Second, in the subgroup analysis of pathology, only triple‐negative and HER2‐positive BC patients were enrolled. Furthermore, all the included studies were written in English which would result in potential publication bias. Finally, future international multi‐center studies with larger sample size were in need to confirm our results.

Nevertheless, the present meta‐analysis was performed at an appropriate time as an adequate number of studies with sufficient data in a large patient cohort investigating the prognostic effect of NLR and PLR for BC patients have been accumulated, allowing evaluation through meta‐analysis. A meta‐analysis is considered to be a statistical inspection of scientific studies which is associated with higher evidence level than the individual studies themselves.^66^ Subgroup analyses were performed to minimize heterogeneity stemming from different BC‐specific subtypes, optional cut‐off values, geographical regions, and tumor stages.

In conclusion, this study suggested that elevated NLR and PLR were associated with poor OS as well as high risk of recurrence for BC patients. Subgroup analyses confirmed negative prognostic value of NLR and PLR for HER2‐positive BC patients. As easily accessible parameters, NLR and PLR should be identified as useful biomarkers in the management of BC.

## CONFLICT OF INTEREST

The authors declare that there are no conflict of interest.

## AUTHOR CONTRIBUTIONS

Wanying Guo and Miao Deng contributed to the designation of this study. Wanying Guo and Xin Lu contributed to literature research. Qipeng Liu and Ting Zhang contributed to data extraction. Xin Lu, Peng Li and Weiqiang Qiao performed the statistical analysis. All the authors participated in drafting the manuscript.
