# Supplementary material for: Prognostic value of neutrophil‐to‐lymphocyte ratio and platelet‐to‐lymphocyte ratio for breast cancer patients: An updated meta‐analysis of 17079 individuals
Source: Cancer Med. 2019 Jun 13;8(9):4135–48. doi: 10.1002/cam4.2281 (PMC6675722; doi:10.1002/cam4.2281)
Supplement: Supplementary file 1 [file CAM4-8-4135-s001.docx]

Supplementary table 1. Summary of inclusion, exclusion criteria and risk of bias.

|  | Inclusion criteria | Exclusion criteria | Study participation | Study attrition | Prognostic factor measurement | Outcome measurement | Study confounding | Statistical analysis and reporting |
| --- | --- | --- | --- | --- | --- | --- | --- | --- |
| Allan et al. 2016 | Patients diagnosed with invasive, non-metastatic breast cancer | Patients with hematological disorders, corticosteroid use or any acute or chronic inflammatory disease | − | − | − | − | − | − |
| Asano et al. 2015 | Patients with resectable, early-stage breast cancer diagnosed as stage IIA, IIB, or IIIA were treated with neoadjuvant chemotherapy | Patients unsuitable for neoadjuvant chemotherapy | ± | − | − | ± | ± | − |
| Asano et al. 2016 | Patients with resectable, early-stage breast cancer diagnosed as stage IIA, IIB, or IIIA were treated with neoadjuvant chemotherapy | Patients unsuitable for neoadjuvant chemotherapy | ± | − | − | ± | ± | − |
| Azab et al. 2013 | Patients diagnosed with breast cancer, with data on complete blood count including leukocyte differential, before initiating any chemotherapy | Patients without any blood count data prior to chemotherapy, presence of active infection, presence of coexisting hematological malignancies or other hematological disorders, autoimmune disorders, and patients on recent steroid therapy | − | − | − | ± | ± | ± |
| Blanchette et al. 2018 | Patients with metastatic HER2-positive breast cancer treated with trastuzumab | Patients with any second malignancy diagnosis, or who were male | ± | − | − | ± | ± | − |
| Bozkurt et al. 2015 | Patients who were diagnosed with primary TNBC and had completed all phases of their primary treatment for the disease | Patients with ductal carcinoma in situ, with or without microinvasion, and patients with incomplete pathological or laboratory results; patients with stage IV breast cancer or inflammatory breast cancer and patients who were diagnosed preoperatively with systemic inflammatory or chronic diseases | − | ± | − | − | ± | − |
| Chae et al. 2018 | Patients with triple-negative breast cancer who underwent neoadjuvant chemotherapy and subsequent breast surgery | Previous treatment for contralateral breast cancer, distant metastasis at initial diagnosis, and a diagnosis of ductal carcinoma in situ | − | − | − | − | ± | − |
| Chen et al. 2016 | Female aged 18 to 70, whose expected survival time was more than 12 months; clinical stage II or III; diagnosed with primary breast cancer by core needle biopsy before NAC; received 3 cycles or more than 3 cycles of NAC after diagnosis and underwent curative-intent surgery | Patients with ductal carcinoma in situ with or without microinvasion, patients with missing information on pathologic or laboratory results, and patients who were lost to follow-up | ± | − | − | − | ± | − |
| Cho et al. 2018 | Patients who underwent surgery for the treatment of invasive breast cancer | Patients who received  neoadjuvant chemotherapy | − | − | − | − | ± | − |
| Cihan et al. 2014 | Patients with breast cancer | Patients with missing data  and those lost in follow-up | ± | − | − | − | ± | ± |
| Dirican et al. 2015 | Patients diagnosed with breast cancer | Patients with active infection active bleeding, blood transfusion within the last 3 months, chronic inflammatory or autoimmune disease, or steroid treatment | − | − | − | − | ± | − |
| Ferroni et al. 2018 | Age above 18 years, an Eastern Cooperative Oncology Group (ECOG) performance status ≤2 and adequate hematological, hepatic and renal functions | Concurrent infectious, inflammatory or autoimmune diseases | ± | − | − | − | ± | ± |
| Forget et al. 2014 | Breast cancer patients undergoing breast cancer surgery | Cancer in the past 5 yr (excluding cutaneous basocellular and in situ uterine cervix carcinomas), previous ipsilateral, and/or non-curative surgery; incomplete medical charts | − | − | − | − | ± | − |
| Gunduz et al. 2015 | Patients who received adjuvant trastuzumab for early and locally advanced breast cancer | Not available | ± | − | − | ± | − | − |
| Hernandez et al. 2017 | Breast cancer patients who were treated with chemotherapy before surgical treatment | Patients with infectious diseases, haematological diseases or undergoing treatment with immunomodulatory drugs; patients with disseminated disease from diagnosis (stage IV of the TNM system) and those with local tumour recurrence or new onset of breast cancer who were also treated with neoadjuvant chemotherapy | ± | − | − | − | − | − |
| Hong et al. 2016 | Patients who were diagnosed with primary invasive breast cancer and had received all phases of treatment | Breast cancer in situ with or without microinvasion, stage IV breast cancer, patients who received neoajuvant chemotherapy, clinical evidence of acute infection, chronic inflammatory or autoimmune diseases, presence of hematological disorders, end stage renal disease, and presence of any other malignancies | − | − | − | − | ± | − |
| Iwase et al. 2017 | Patients who had breast cancer recurrence during the follow‑up period after surgery | Patients who already had a metastatic site at the time of initial diagnosis | ± | ± | − | ± | ± | ± |
| Jia et al. 2015 | Breast cancer patients with a complete blood count and a leukocyte differential count before initiating any treatment and the medical records were available for these patients | Patients who had received any treatment before surgery or neoadjuvant chemotherapy or who had metastatic disease; bilateral breast cancer, male breast cancer, inflammatory breast cancer, and those on long term corticosteroids therapy | − | − | − | − | ± | ± |
| Koh et al. 2014 | ER positive and/or PR positive and  HER2-negative breast cancer began neoadjuvant chemotherapy, followed by definitive surgical resection | No one was excluded | − | − | − | − | − | − |
| Koh et al. 2015 | Pre-operative blood count results (counts taken as part of pre-operative assessment) or counts taken before systemic therapy/radiotherapy in those who did not undergo surgery | Patients with unknown ER, PR or HER2 status, as well as those with equivocal HER2 status without fluorescence in situ hybridisation | − | ± | − | − | − | − |
| Krenn-Pilko et al. 2014 | Patients with histologically confirmed breast cancer | Not available | ± | − | − | − | ± | − |
| Krenn-Pilko et al. 2016 | Patients with histologically confirmed breast cancer | Not available | ± | − | − | ± | ± | ± |
| Lee et al. 2018 | Triple-negative breast cancer patients were pathologically diagnosed as stage I to III and received adequate local, systemic treatment; adequate bone marrow function without hematologic disease; patients who regularly followed up | Not available | ± | ± | − | − | ± | − |
| Limori et al. 2018 | Patients who underwent endocrine therapy as initial drug therapy for stage IV breast cancer | Not available | ± | ± | − | ± | ± | ± |
| Liu et al. 2016 | Histologically confirmed HR- nonmetastatic breast cancer, with pretreatment data of differential blood counts, and no history of inflammatory and immune disease, diabetes, hypertension, metabolic syndrome, coronary artery disease, renal disease, and hematological disease | Patients with metastatic and inflammatory breast tumors and infectious diseases | ± | − | − | − | − | ± |
| Mando et al. 2018 | Nonmetastatic breast cancer patients underwent surgery | Not available | − | − | − | − | ± | ± |
| Miyagawa et al. 2018 | Patients with metastatic breast cancer treated with eribulin or nab-paclitaxel | Patients who received combination therapy with other chemotherapy agents, anti- HER2 therapy, or endocrine therapy | ± | ± | − | ± | − | − |
| Nakano et al. 2014 | Patients with operable breast cancer | Patients with ductal carcinoma in situ with or without microinvasion and patients lacking pathological or laboratory information; patients who had received chemotherapy or immunosuppressive drugs | − | − | − | ± | − | ± |
| Orditura et al. 2016 | Female patients with histologically proven  early (T1–2, N0–1, non-metastatic) breast cancer | Patients whose clinical records were lacking data relevant to this study or because of preoperative chemotherapy, and 24 patients with abnormal white cell counts possibly due to concomitant infectious diseases, autoimmune diseases, or other recognisable inflammatory conditions, | ± | − | − | − | ± | − |
| Pistelli et al. 2014 | Patients who were diagnosed and completed the treatment of invasive breast cancer | Patients with ductal carcinoma in situ with or without micro-invasion and patients with lack of information on pathologic or laboratory results; patients with stage IV breast cancer or inflammatory breast cancer, patients who were diagnosed preoperatively with systemic inflammatory or chronic disease | ± | − | − | − | ± | − |
| Qiu et al. 2018 | Non-metastatic triple-negative breast cancer patients diagnosed with invasive breast cancer | Patients with stage IV or inflammatory breast cancer, as well as those who had been diagnosed with systemic inflammatory or chronic disease | ± | − | − | − | ± | − |
| Takeuchi et al. 2017 | Breast cancer patients who underwent surgery | Patients with distant metastases at initial presentation, carcinoma in situ, bilateral breast carcinoma, and male breast carcinoma; patients with comorbidities that affected serum CRP levels, including infection, collagen disease, and liver cirrhosis; and patients with incomplete laboratory data | − | − | − | − | ± | − |
| Takuwa et al. 2018 | Patients with histologically or clinically confirmed metastatic breast cancer | Not available | ± | − | − | ± | ± | ± |
| Templeton et al. 2018 | Patients who participated in the randomized phase 3 GEICAM/9906 study | Patients without information on neutrophils and leukocytes and patients with baseline leukocyte counts > 15 × 109/L | − | − | − | ± | ± | − |
| Ulas et al. 2015 | HER2-positive early breast cancer patients who underwent resection and received adjuvant trastuzumab | Patients who had metastasis at the time of diagnosis, those with contralateral breast cancer or cancer other than breast cancer, those who received neoadjuvant treatment, who had evidence of clinically active infection, those with hematological, chronic inflammatory or autoimmune diseases, and patients who were on active steroid treatment | − | − | − | ± | ± | ± |
| Vernieri et al. 2018 | Metastatic triple negative breast cancer received platinum-containing ChT as their first- or second-line treatment | Patients whose blood parameters were measured more than one month before ChT initiation, or after having received the first dose of platinum-based ChT | ± | ± | − | − | ± | − |
| Wariss et al. 2017 | Women with breast cancer diagnosed and treated in a single referral center | Women under the age of 18 years | ± | ± | − | ± | ± | − |
| Yao et al. 2014 | Female patients who were diagnosed with primary breast cancer and who were treated with surgery | Patients with any inflammatory signs or conditions, hematological disease, coronary artery disease, end-stage renal disease, heart failure, cerebrovascular disease, peripheral arterial disease, or a lack of information pertaining to pathologic or laboratory results | − | − | − | − | − | ± |
| Zhang et al. 2016 | Patients diagnosed with breast cancer, with data on CBC, including RCIs and leukocyte differential count, prior to initiating any chemotherapy | Patients without any blood count data prior to chemotherapy, presence of active infection, presence of coexisting hematological malignancies or other hematological disorders, autoimmune disorders, and patients on recent steroid therapy | − | ± | − | ± | ± | − |

The risk of bias for each domain was rated as low (−), moderate (±) or high (+).
